# The role of timely assessment of appropriate care in reducing greenhouse gas emissions in intensive care medicine

**DOI:** 10.1016/j.aicoj.2026.100162

**Published:** 2026-09-17

**Authors:** Paul-Simon Pugliesi, Lucas Mathey, Jean-Philippe Rigaud, Eliott Teston, Dima Siblani, Romain Ter Schiphorst, Marie Labruyère, Caroline Abdul Malak, Serge Ribes, Christine Binquet, Catherine Lejeune, Thomas Maldiney, Jean-Pierre Quenot

**Affiliations:** a Service Réanimation polyvalente, Centre Hospitalier William Morey, 71100 Chalon sur Saône, France; b Service Développement durable, Centre Hospitalier William Morey, 71100 Chalon sur Saône, France; c Service de Médecine Intensive-Réanimation, Centre Hospitalier de Dieppe, 76200 Dieppe, France; d Espace de Réflexion Éthique de Normandie, CHU de Caen, 14033 Caen Cédex, France; e Lipness Team, Translational Research Center in Molecular Medicine– INSERM Joint Research Unit (CTM-UMR1231), University of Burgundy, 21000 Dijon, France; f Service Médecine Intensive Réanimation, CHU Dijon Bourgogne, 21000 Dijon, France; g Université Bourgogne Europe, CHU Dijon Bourgogne, Centre d'Investigation Clinique, module épidémiologie clinique, INSERM, CIC 1432, Dijon, France

**Keywords:** Intensive care, Ethics, Sustainability, Greenhouse gas emissions

## Abstract

**Background:**

Our era is characterized by the significant impact of anthropogenic activity, resulting in the paradox of short-term benefits, such as improved health, alongside potentially devastating long-term consequences for our species and the living world. Critical care, by attempting to preserve human lives through the massive mobilization of human, energy, and material resources, is a quintessential example of this paradox. It is therefore an ideal setting to explore the necessary compromise between care provided for the benefit of patients and the requirement to protect future ecological viability. The objective of our study was twofold: first, to evaluate whether conducting ethical reflections that lead to the early limitations, withdrawal, or non-initiation of certain invasive treatments to avoid disproportionate care would result in a reduction of greenhouse gas (GHG) emissions associated with critical care stays; and second, to quantify such a reduction.

**Results:**

We conducted a retrospective study analyzing both the medical activity and GHG emissions of a French critical care department throughout the year 2023. Our analysis found that one day of hospitalization required an average atmospheric emission of 194 kg CO_2_ equivalent (CO_2_eq) in the Intensive Care Unit (ICU) and 160 kg CO_2_eq in the Intermediate Care Unit (IMCU). Procurement (medications, medical devices, medical equipment) and transport (employees and patients) represented the primary sources of GHG emissions. The analysis of medical activity covered 828 patient stays, corresponding to 2913 patient-days in the ICU and 1225 in the IMCU. The overall mortality rate in critical care was 17% (31% in ICU, 5% in IMCU). In the subgroup for which a total or partial limitation of invasive care was decided within 48 h of admission (240 stays or 29%), each stay required an average atmospheric emission of 586 kg CO_2_eq, compared to 1055 kg CO_2_eq for the remainder of the cohort (*p* = 0.004).

**Conclusion:**

An ethical approach consisting of a timely assessment of the appropriate level of care for each patient, with the goal of avoiding disproportionate care, appears to significantly reduce GHG emissions per stay. This approach could constitute a vital component of sustainability within critical care services.

## Background

Intensive care is characterized by a large number of healthcare professionals utilizing significant material resources for a small proportion of patients. These characteristics generate very high economic and environmental costs related to life-sustaining therapies (e.g., mechanical ventilation, renal replacement therapy, circulatory support), etiological investigations, pharmacological or non-pharmacological therapies, and continuous monitoring devices for patient vital signs [[1], [2], [3]]. Concomitantly, critical care management must remain proportional to the patient's clinical status, prior history, and long-term prognosis. This care plan should be continuously re-evaluated through a collaborative, collegial process involving the healthcare team, the patient's advance directives, and discussions with the next of kin [4]. Each clinical situation remains unique, requiring interventions to be tailored to both clinical trajectory and patient preferences.

In critical care, the combination of high-cost interventions—both economically and environmentally—and profound human stakes raises a major ethical dilemma centered on the fair allocation of resources. Addressing this challenge requires anchoring intensive care practices in the principles of distributive justice and civic solidarity, with the ultimate goal of ensuring equity in patient care now and in the future [5].

From this perspective, the proactive ethical determination of care goals could address this issue and enable intensive care stakeholders to engage in an approach that aligns with the contemporary challenge of sustainability [6,7]. As has been shown, early identification of situations involving non-beneficial or disproportionate intensive care remains a major challenge in avoiding non-beneficial treatment and care fragmentation [8]. Such a proactive approach, while serving individuals treated in the present, would also have the positive byproduct of preserving the future—specifically by reducing the environmental impact of critical care stays through the forgoing of certain technical interventions and the reduction of lengths of stay.

To explore these perspectives, this study presents a retrospective description of a critical care department’s clinical activity alongside an inventory of its associated greenhouse gas (GHG) emissions. We hypothesize that anticipating proportionate, individualized care ensures optimal patient management while at the same time contributing to healthcare sustainability. The overarching goal was to evaluate and quantify whether ethical considerations leading to the early withholding or withdrawal of certain invasive treatments result in a reduction of GHG emissions for the stays in question.

## Methods

### Study design and population

We conducted a single-center, retrospective study involving adult patients admitted between 1 January and 31 December 2023, to the critical care department of the Chalon-sur-Saône Hospital, which is divided into two units: the intensive care unit (ICU) and the intermediate care unit (IMCU).

### Data collection

The following stay-related data were collected: length of stay in the critical care department, unit (IMCU or ICU), admission category (medical, scheduled surgery, emergency surgery, trauma, or psychiatry), reason for admission, Simplified Acute Physiology Score II (SAPS II) score, and use of life-sustaining therapies (vasopressors, renal replacement therapy, mechanical ventilation, non-invasive ventilation, and high-flow nasal oxygen). We also recorded whether an admission was considered potentially inappropriate (as judged by the patient, the referring medical team, or the critical care team during a multidisciplinary ethical review documented in the medical record) [9]. Additionally, data were collected on whether a treatment-limitation plan (a decision to withhold specific invasive procedures) was established within 48 h of admission, as well as mortality during the stay and within 6 months following admission.

The following patient clinical data were also collected: age, sex, body mass index (kg/m²), presence of a non-curable malignancy, an advanced respiratory disease (long-term oxygen therapy, bilevel positive airway pressure ventilation, or Forced expiratory volume (FEV1) <1 L), a chronic kidney disease (eGFR <60 mL/min/1.73 m²), an end-stage renal disease (requiring renal replacement therapy), a severe heart disease (Left ventricular ejection fraction (LVEF) <30%, Pulmonary hypertension (PH) >50 mmHg), diabetes, and cirrhosis.

#### Subgroup

Patients were categorized into the Early Ethical Component (EEC) subgroup if their stay involved ethical considerations aimed at adjusting the level of care within the first 48 h of admission. This subgroup comprised patients with early treatment-limitation plans established at admission and/or those whose admission was deemed potentially inappropriate within the first 48 h, including patients who met both criteria. All other patients, including those with delayed treatment-limitation plans decided after 48 h, were included in the non-EEC comparison group.

#### Greenhouse gas emissions inventory

The estimation of GHG emissions was carried out following the methodology recommended by the French Agency for Ecological Transition (ADEME) [10] in accordance with the ISO 14064-1 standard. Additionally, we relied on the guidelines for performing GHG emissions assessments for healthcare facilities published by ADEME (2020 version) [11].

### Assessment scopes

The organizational scope encompassed the entire critical care department, including all equipment and facilities under its control. The operational scope integrated all physical flows (i.e., activity data measured in physical units such as kilowatt-hours, kilograms, or kilometers) required for the standard functioning of the department: direct energy and water consumption, staff and patient transportation, all purchases of goods and services (food, pharmaceuticals, medical devices, imaging, and laboratory tests), fixed assets (buildings, parking lots, medical equipment), and waste disposal and treatment. Fig. 1 illustrates the study scopes and the mapping of the analyzed physical flows.

**Fig. 1:**
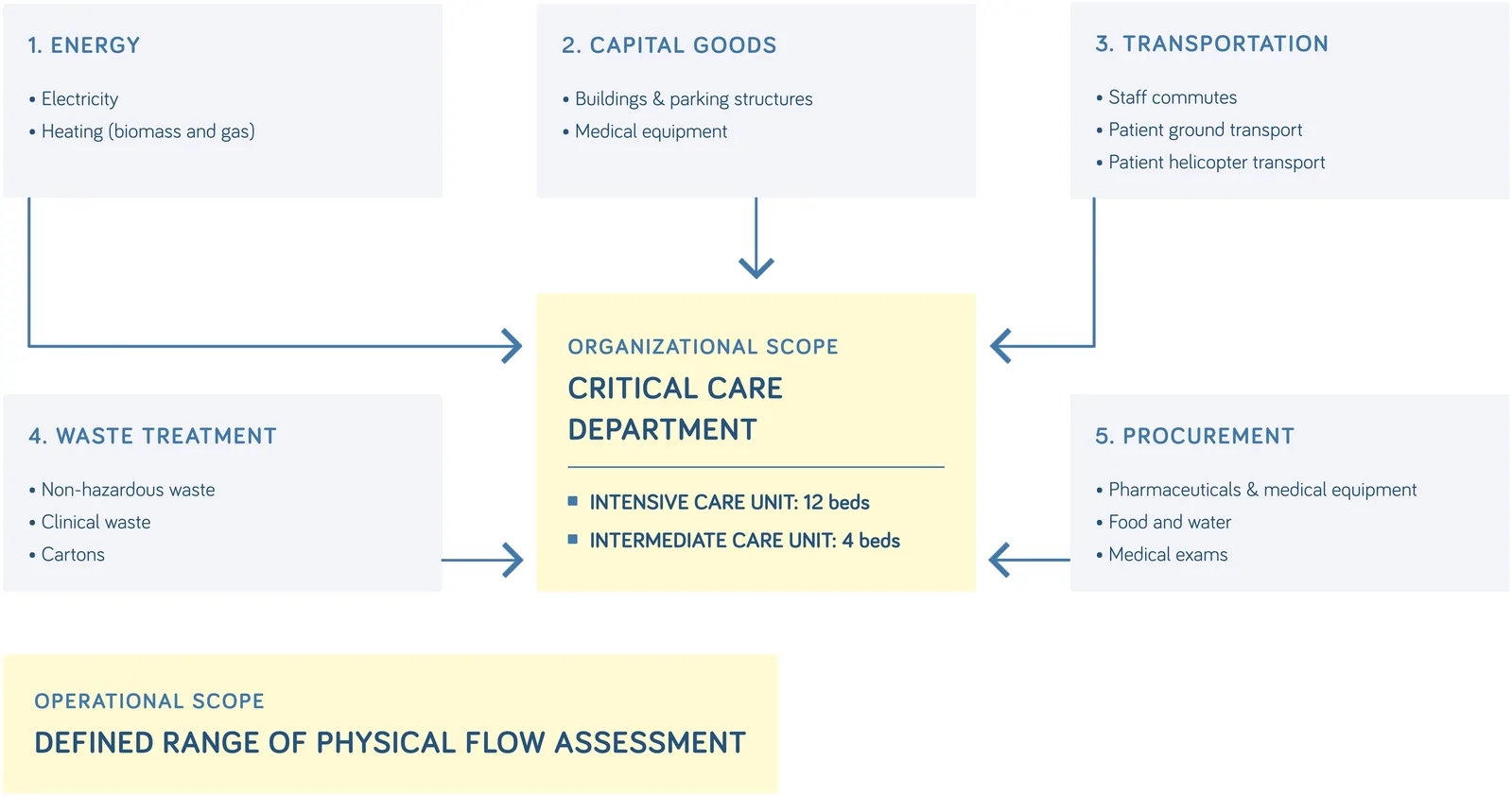
Organizational and operational scope of the present assessment of GHG emissions at Chalon-sur-Saône Hospital Center for the year 2023.

### GHG emissions calculation

Estimating the GHG emissions induced by the activity of the critical care department required two steps: the quantification of physical flows and the calculation of emissions attributable to each flow.

Each physical flow included in the operational scope was quantified with the assistance of resource personnel. Electricity and water consumption were measured directly via dedicated meters; staff commutes were quantified through a specific questionnaire; and patient transportation was measured using data provided by the Emergency Medical Services (SAMU) dispatch. Support services provided data on procurement flows, services, and capital investments (either in quantity or monetary value). Finally, waste production was estimated through regular weighing.

Each quantified physical flow was converted into GHG emissions using emission factors (i.e., estimates of GHG emissions embedded in one unit of physical flow). These factors were either provided by resource personnel or sourced from established databases and scientific literature (see Supplementary Material). Summing GHG emissions linked to each physical flow allows for the estimation of the overall carbon (and thus climate) footprint of the department, expressed in metric tons of CO_2_eq (Fig. 2).

**Fig. 2:**
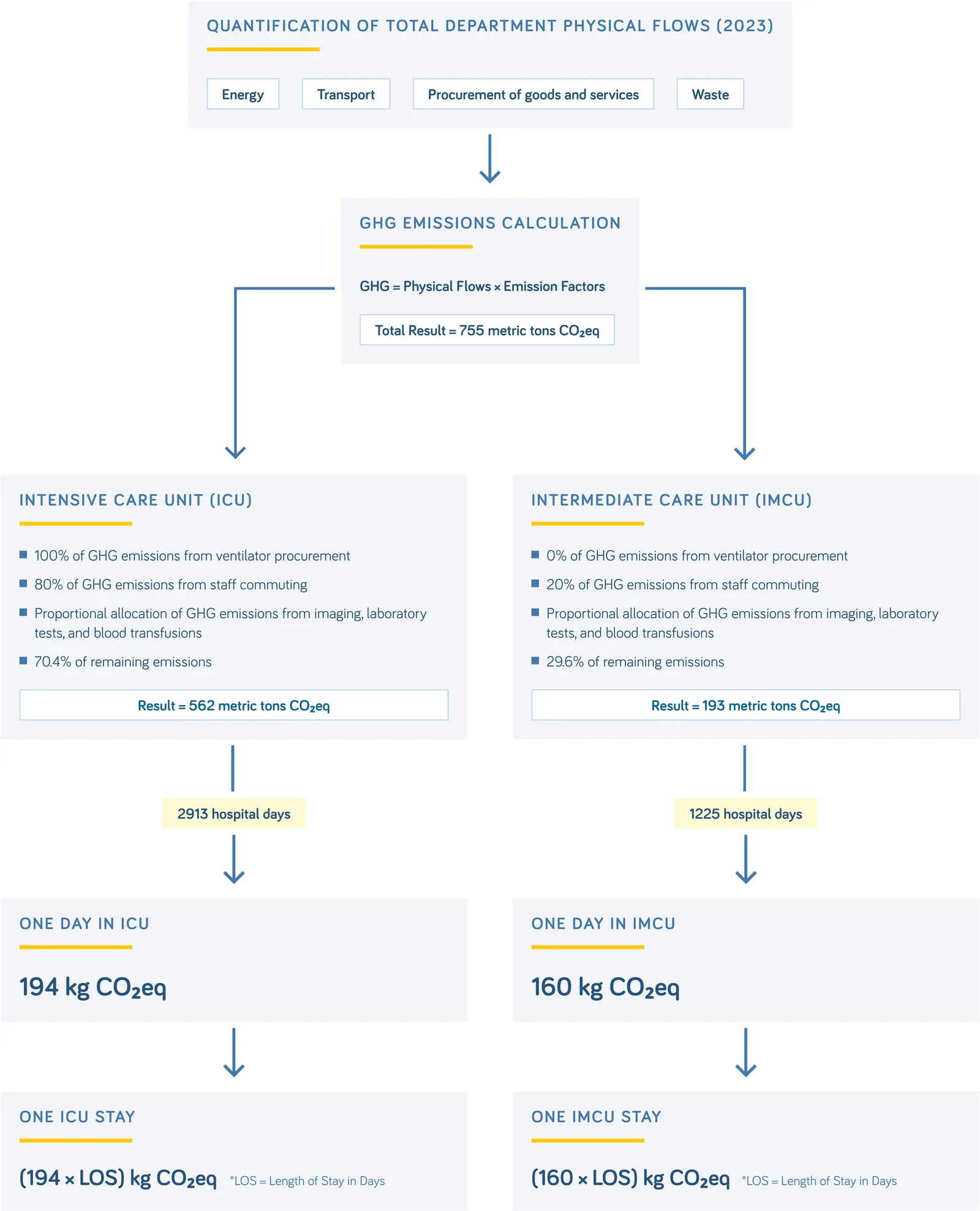
Method for GHG emission calculation and allocation.

### Data processing and allocation

Regarding the allocation of emissions between the IMCU and the ICU: emissions related to the purchase of ICU ventilators were attributed solely to the ICU. Staff commuting emissions were allocated 80% to the ICU and 20% to the IMCU, in accordance with the staff distribution between the two units. Emissions related to imaging, laboratory tests, and blood product transfusions were distributed between the two units based on the specific location of the patients who received them. Whenever location-specific data were unavailable for shared baseline flows, these residual emissions were allocated according to clinical activity levels, based on the annual number of patient-days in each unit. Once all emissions were distributed across the two subunits, the total was divided by the number of patient-days to obtain an estimated GHG emission rate per day of hospitalization in both the ICU and the IMCU. Emissions for each individual stay were calculated by multiplying the daily emissions by the length of stay (Fig. 2).

### Statistical analysis

Qualitative variables are expressed as numbers and percentages, while quantitative variables as mean ± standard deviation. For subgroup analysis, comparisons of qualitative variables were performed using the Chi-squared test, while quantitative variables were compared using the Wilcoxon rank-sum test (univariate analysis).

## Results

### Clinical data

In 2023, the critical care department at Chalon-sur-Saône Hospital admitted 828 adult patients, of whom 384 (46%) were admitted to the ICU and 444 (54%) to the IMCU. All patients were included in the analysis. The mean length of stay was 7.6 (±9.7) days in the ICU and 2.8 (±2.7) days in the IMCU. The entire cohort represented a total of 4,138 patient-days, of which 2,913 (70.4%) occurred in the ICU. Detailed characteristics of the cohort are presented in Table 1.

**Table 1:** Patient characteristics and stay data included in the analysis cohort (Chalon sur Saône Hospital Critical Care Department, N = 828).

Patient characteristics			
	IMCU (n = 444)	ICU (n = 384)	Total critical care (n = 828)
Age (years)	63 (±18)	67 (±14)	65 (±17)
Male sex	237 (53,4%)	289 (75.3%)	526 (63.5%)
BMI a (kg/m^2^)	26.7 (±7)	27.2 (±6.5)	26.9 (±6.9)
Advanced respiratory disease*	32 (7.2%)	28 (7.3%)	60 (7.2%)
Severe heart disease**	22 (4.9%)	30 (7.8%)	52 (6.3%)
CKD ***	42 (9.4%)	51 (13%)	93 (11.2%)
ESRD	15 (3.4%)	16 (4.2%)	31 (3.7%)
Non-curable malignancy	38 (8.6%)	46 (11.9%)	84 (10.1%)
Diabetes mellitus	110 (24.8%)	112 (29.1%)	222 (26.8%)
Cirrhosis	20 (4.5%)	21 (5.5%)	41 (4.9%)

Stay characteristics			
	IMCU (n = 444)	ICU (n = 384)	Total critical care (n = 828)
Admission type			
Medical emergencies	254 (57.2%)	314 (81.8%)	568 (68.6%)
Scheduled surgeries	80 (18%)	4 (1%)	84 (10.1%)
Emergency surgeries	40 (9.2%)	41 (10.6%)	81 (9.8%)
Trauma	41 (9.2%)	13 (3.4%)	54 (6.5%)
Psychiatric emergencies	23 (5.2%)	12 (3.1%)	35 (4.2%)
Others	6 (1.4%)	0	6 (0.7%)
SAPS II^b^ (mean ± SD)	31 (±13)	53 (±33)	41.6 (±26.8)
Life-sustaining therapies			
Vasopressors	9 (2%)	246 (64%)	255 (30.8%)
Invasive mechanical ventilation	14 (3%)	234 (60%)	248 (29.9%)
Non-invasive ventilation	33 (7.4%)	50 (13%)	83 (10%)
High-flow nasal oxygen	8 (1.8%)	21 (5.5%)	29 (3.5%)
Renal replacement therapy	10 (2.2%)	90 (23%)	100 (12%)
Potentially inappropriate admissions✝	72 (16%)	27 (7%)	99 (11.9%)
Early treatment-limitation plans ✝✝	92 (20.7%)	104 (27%)	196 (23.7%)
Length of stay (M ± SD)	2.8 (±2.7)	7.6 (±9.7)	5 (±7.3)
Critical care mortality	24 (5.4%)	119 (30.9%)	143 (17.2%)
6-month mortality	87 (19.6%)	176 (45.8%)	263 (31.7%)

* Long-term oxygen therapy or non-invasive ventilation, FEV1 < 1 L.

** Left ventricular ejection fraction (LVEF) <30% or pulmonary artery pressure PAP >50 mmHg.

*** Chronic Kidney Disease: Creatinine clearance <60 ml/min/1.73 m^2^ (CKD-EPI).

✝ Admission deemed as such by the patient, the referring medical team, and/or the clinical consensus of the critical care multidisciplinary team.

✝✝ Early decision (<48 h) to limit access to specific invasive procedures on the grounds that their use would constitute non-beneficial or disproportionate care.

a = Body Mass Index.

b = Simplified Acute Physiology Score II.

The mean age of included patients was 65 (±17) years, and 63.5% were male. Overall, 68.6% of patients were admitted for a medical emergency (as opposed to surgical, psychiatric, or trauma emergencies); this figure rose to 81.8% for ICU admissions. Furthermore, 99 admissions (11.9%) were classified as potentially inappropriate, based on the judgment of the patient, the referring medical team, and/or the consensus opinion of the critical care medical team.

An early treatment-limitation plan was established for 196 patients (23.7%). In total, 240 stays (29%) involved early ethical considerations that led to a partial or total limitation of the level of care. This figure of 240 represents the sum of the 99 inappropriate admissions and the 196 treatment-limitation decisions, following the removal of duplicates, as 55 admissions fell into both categories.

The overall mortality rate within the department was 17.2% (30.9% in the ICU and 5.4% in the IMCU). Six months after admission, 263 patients (31.7%) had died. The 6-month mortality rates were 45.8% and 19.6% for the ICU and the IMCU, respectively.

### GHG emissions

The estimated GHG emissions associated with the activity of the Chalon-sur-Saône critical care department in 2023 totaled 755 metric tons of CO_2_eq. Of this total, 562 tCO_2_eq (74%) were attributed to the ICU and 193 tCO_2_eq (26%) to the IMCU. The breakdown of emissions by category and sub-item is presented in Table 2.

**Table 2:** Breakdown of estimated GHG emissions for the critical care department of Chalon sur Saône Hospital in 2023.

Physical flows/categories	Quantities	GHG emissions (kgCO_2_eq)	Share of total department emissions (%)
Engergy			
Electricity for ventilation and air filtration	282,258 kW h	10,662	1.4%
Electricity for air conditioning	32,807 kW h	1,239	0.2%
Electricity for heating (heat pumps)	7141 kW h	270	0.0%
Other electricity (lighting, medical machines)	301,390 kW h	11,385	1.5%
Heating (biomass and fossil gas)	61,286 kW h	7,477	1%
**Subtotal**	-	**31,033**	**4.1%**
Transportation			
Staff commuting	683,302 km	74,029	9.8%
Patient ground transportation	26,794 km	5,841	0.8%
Kerosene for helicopter transport	18,480 L	61,389	8.1%
**Subtotal**	-	**141,260**	**18.7%**
Procurement of goods and services			
Pharmaceutical products	432,046 €	216,023	28.6%
Medical devices	323,856 €	102,015	13.5%
Blood transfusions	482	3,644	0.5%
Imaging exams	2,827	4,685	0.6%
Laboratory tests	19,202	1,592	0.2%
Meals	4387	8,949	1.2%
Water (production and treatment)	400 m^3^	3,943	0.5%
Services (maintenance and laundry)	104,578 €	19,826	2.6%
Office equipment	6 942 €	3,521	0.5%
**Subtotal**	-	**364,198**	**48.2%**
Waste treatment			
Non-hazardous waste	22,000 kg	9,702	1,3%
Clinical waste (infectious risk)	7500 kg	7,005	0.9%
Cardboard	4000 kg	188	0.0%
**Subtotal**	-	**16,895**	**2.2%**
Fixed assets (Capital goods)			
Buildings	2012 m^2^	44,264	5.9%
Parking infrastructure	1798 m^2^	13,123	1.7%
Mattress rentals	117,696 €	23,539	3.1%
Hemodialysis machines	32,000 €	2,240	0.3%
ICU ventilators	277,994 €	64,865	8.6%
Other biomedical equipment	229,000 €	53,433	7.1%
**Subtotal**	-	**201,464**	**26.7%**
Totals			
**Total for 2023**	-	**754,850**	**100%**
**Per ICU patient-day**	-	**194**	-
**Per IMCU patient-day**	-	**160**	-

Physical flows are expressed in specific units: energy in kilowatt-hours (kWh), distances in kilometers (km), fuel quantities in liters (L), monetary flows in euros (€), water in cubic meters (m³), and surface areas in square meters (m²); counts/quantities are unitless. The calculation of GHG emissions associated with each physical flow was performed using emission factors (see Supplementary Material). Estimated emissions for a single ICU or IMCU patient-day represent the ratio between the total emissions for each unit and the total number of patient-days recorded in that unit during the year.

Pharmaceuticals accounted for the largest share of emissions, totaling 216 tCO_2_eq (29%). This was followed by purchases of biomedical equipment (e.g., ventilators), representing 118 tCO_2_eq (16%), medical devices (102 tCO_2_eq, 14%), staff commuting (74 tCO_2_eq, 10%), and patient helicopter transport (61 tCO_2_eq, 8%). The critical care department’s energy consumption (electricity and heating) was estimated at approximately 670 MW h for the year, equivalent to 1835 kW h per day or 810 kW h per stay. This energy use was associated with the emission of 31 tCO_2_eq, representing 4% of the total. Waste treatment accounted for only 17 tCO_2_eq (2%) of total emissions.

We have estimated that one patient-day was responsible for an average emission of 194 kg CO_2_eq in the ICU and 160 kg CO_2_eq in the IMCU (Fig. 2). For the entire cohort (n = 828), a stay in a critical care unit required the emission of 912 kg CO_2_eq into the atmosphere.

### Subgroup comparison

The proactive assessment of the potential consequences of medical interventions resulted in either a decision to forgo all specific critical care interventions (potentially inappropriate admission) or limit therapy to only specific interventions (treatment-limitation plan) for 240 stays (29%). These cases constituted the EEC group.

This group was compared with the remainder of the cohort (non-EEC group, n = 588). In this second group, initial care decisions did not include an ethical component, either because it was deemed unnecessary or because such an assessment could not be performed for various reasons (e.g., time constraints, oversight, organizational challenges, or lack of clinical context). In this non-EEC group, 69 patients (12%) experienced a delayed treatment-limitation plan later during their ICU stay. Patient and stay characteristics for both subgroups are presented in Table 3.

**Table 3:** Patient and stay characteristics, clinical outcomes, and environmental impact of the EEC and non-EEC subgroups.

Variables	EEC Group (n = 240)	Non-EEC Group (n = 588)	p-value
Patient characteristics			
Mean age (SD)	70.57 (14.84)	60.72 (16.85)	<0.001
Female gender (%)	40.83%	34.69%	0.1
Mean BMI (kg/m^2^)	26.07 (6.85)	27.30 (6.63)	0.08
Non-curable malignancy (%)	21.67%	5.44%	<0.001
Advanced respiratory disease (%)	14.17%	4.42%	<0.001
Severe heart disease (%)	10.42%	4.59%	0.002
CKD (%)	12.92%	10.54%	0.3
Diabetes (%)	28.33%	26.19%	0.5
Cirrhosis (%)	4.58%	5.10%	0.8
Stay characteristics			
Admission to IMCU (%)	55.00%	53.06%	0.6
Admission to ICU (%)	45.00%	46.94%	0.6
Mean SAPS II score	46.6 (18.9)	39.82 (29.0)	<0.001
Mechanical ventilation (%)	25.42%	31.80%	0.069
Duration of mechanical ventilation (days)	3.7 (4.13)	7.60 (9.60)	<0.001
Vasopressors (%)	31.25%	30.61%	0.9
Non-invasive ventilation (%)	15.00%	7.99%	0.002
Renal replacement therapy (%)	10.00%	12.93%	0.2
Mean length of stay (days)	3.29 (3.41)	5.70 (9.42)	0.001
Survival and GHG emissions			
**GHG emissions per stay (kgCO_2_eq)**	**585.9**	**1,055.3**	**0.004**
Critical care unit survival (%)	65.42%	89.80%	<0.001
6-month survival (%)	40.42%	76.53%	<0.001

*Guide*. Patients in the “EEC” group had a mean age of 70.57 years, whereas those in the “non-EEC” group had a mean age of 60.72 years, representing a statistically significant age difference (*p* < 0.001). Quantitative variables are expressed as mean (standard deviation), and the Wilcoxon rank-sum test was used for statistical comparison. Qualitative variables are expressed as percentages, and the Chi-square test was used for comparison.

On average, patients in the EEC group were 10 years older than patients in the non-EEC group, with respective mean ages of 70.5 and 60.7 years (*p* < 0.001). They also presented with a higher burden of comorbidities, including significantly higher rates of advanced respiratory disease (14% vs. 4%, *p* < 0.001), severe chronic heart failure (10% vs. 4%, *p* = 0.002), and a higher prevalence of non-curable malignancies (22% vs. 5%, *p* < 0.001). There were no significant differences between the two groups regarding the frequency of diabetes or cirrhosis.

Regarding critical care stay characteristics, 25% of patients in the EEC group received invasive mechanical ventilation, compared to 32% in the non-EEC group (*p* = 0.069). When invasive ventilation was initiated, its duration was significantly shorter in the EEC group (3.7 vs. 7.6 days; *p* < 0.001). The mean length of stay (LOS) in critical care was also significantly lower in the EEC group (3.3 vs. 5.7 days; *p* = 0.001). Mean SAPS II scores at admission were 46.6 in the EEC group and 39.8 in the non-EEC group (*p* < 0.001), corresponding to a predicted survival rate during the stay of 61% and 76%, respectively. Observed survival in the critical care unit was 65.4% in the EEC group and 90% in the non-EEC group (*p* < 0.001). At six months post-admission, the overall survival rate was 40% and 76% for the EEC and non-EEC groups, respectively (*p* < 0.001).

Finally, the average GHG emissions were 586 kg CO_2_eq per stay in the EEC group compared to 1055 kg CO2eq in the non-EEC group, representing a significant 44.5% reduction in emissions (*p* = 0.004).

## Discussion

### Impact of early ethical considerations with treatment limitation decisions on GHG emissions

The most notable finding of our study concerns the analysis of GHG emissions associated with stays where medical decisions integrated early ethical considerations. These considerations led to the total or partial withdrawal of invasive procedures to avoid non-beneficial treatment. This subgroup comprised 240 stays (29% of the 828 studied) and involved patients who were significantly older (by approximately 10 years) and more likely to have severe comorbidities (such as non-curable malignancies and advanced respiratory diseases). On average, stays in this subgroup resulted in the emission of 586 kg CO_2_eq, compared to an average of 1,055 kg CO_2_eq for the rest of the cohort (*p* = 0.004, Table 3), representing a 44.5% reduction.

This significant result supports the idea that “appropriateness of care” is a fundamental pillar of environmentally sustainable healthcare and reinforces the intuition that early integration of a palliative approach in critical care can have a broader societal impact by preserving the environment and conserving resources [12]. Furthermore, methodological limitations (discussed below) likely minimized this difference. Indeed, the lack of granular tracking of individual physical flows per stay partially masked the fact that the material intensity of care (such as the consumption of pharmaceuticals, medical devices, and energy) was reduced by early decision to forgo certain invasive treatments. Consequently, the difference in GHG emissions highlighted here primarily reflects reduced lengths of stay. To our knowledge, no prior studies have utilized a similar approach. Given the lack of available comparisons and the single-center nature of our work, the robustness of this result cannot be fully measured and warrants confirmation through further research.

### Measurement of GHG emissions

Another key result of this study is the calculation of GHG emissions for a French critical care department (Chalon-sur-Saône Hospital). Annual emissions totaled 755 metric tons of CO_2_eq. When normalized by the number of patient-days, these figures amount to 194 kg CO_2_eq and 160 kg CO_2_eq per day for the ICU and the IMCU, respectively. Although slightly higher, these values are consistent with previously published data, thereby reinforcing the validity of our results. In 2018, a study by McGain et al. [13] involving 20 septic shock patients in the USA and Australia reported results ranging from 88 to 178 kg CO_2_eq per patient-day. In 2022, Prasad et al. [1] conducted a study in a US critical care unit where GHG emissions were approximately 140 kg CO_2_eq per patient-day. It is important to note that these studies did not consider the same operational scope as our study when making their evaluations, which could explain the minor differences observed.

However, the single-center nature of our work limits its generalizability. In particular, the “transportation” and “energy” categories of the calculation were influenced by local specificities. For example, staff commuting accounts for nearly 10% of total emissions, as employees predominantly use internal combustion engine vehicles. This emission category would likely be lower in urban areas where public and low-carbon transport are more developed. Similarly, the fact that Chalon-sur-Saône Hospital uses a helicopter for patient transport increases GHG emissions, accounting for over 8% of the total emissions on its own (despite a low number of trips). Regarding energy, it is noteworthy that the department’s considerable electricity consumption (approximately 600 MW h over the year) is associated with just over 20 metric tons of CO_2_eq (3% of the total). This result is the direct consequence of the very low carbon intensity of the French electricity mix [14].

Furthermore, one may question whether local epidemiology influences medical activity and, consequently, GHG emissions. Patients included in our study had a mean SAPS II score of 41.6 (±26.8), an in-unit mortality rate of 17.2%, and a mean LOS of 5 (±7.3) days. These results are relatively comparable to French national averages recently published via national database extractions [15]. Therefore, we can reasonably assume that local epidemiological specificities did not constitute a major bias in extrapolating our findings.

Additionally, our calculation methodology does not perfectly isolate variable operational emissions from fixed structural overheads, as a baseline fraction of energy consumption remains independent of daily occupancy. However, this limitation is mitigated by agile critical care management—where seasonal adjustments allow for the partial modulation of structural resources when activity declines.

Finally, it must be noted that we were unable to precisely study the GHG emissions associated with each individual admission. In our study, the emissions attributed to a stay correspond solely to the product of the length of stay and the average daily emission value for the ICU (194 kg CO_2_eq) or the IMCU (160 kg CO_2_eq). However, these two values are averages derived from a department-wide global calculation (Fig. 2). The actual emission values for each stay likely vary significantly depending on the reasons for admission, particularly regarding the use of pharmaceuticals (i.e., sedatives, neuromuscular blockers, antibiotics, vasopressors), medical devices, and biomedical equipment. This represents a limitation of our study that warrants further research.

### Critical care in the anthropocene

Modern healthcare systems emerged and rapidly expanded during the Anthropocene, a geological epoch characterized by human activity becoming the dominant force altering conditions for life on the planet [[16], [17], [18], [19]]. While this unprecedented historical trajectory has driven an exponential increase in global life expectancy [20], it now faces critical planetary and environmental boundaries that demand a profound reassessment of our care models [[21], [22], [23]].

Following in the footsteps of Professor Jean Hamburger (1909–1992), the founder of the concept of medical intensive care in France, we believe that physicians must seek a “compromise… from which the individual emerges intact, yet which allows for the flourishing of social imperatives and a collective organization that protects the human species” [24, p. 154, translation by the author]. In critical care, we hypothesize that this compromise lies in a medical approach consisting of the timely identification of situations in which the use of intensive care interventions may result in non-beneficial treatment for the patient. We believe that this approach—anticipating the consequences of invasive procedures and making early decisions regarding their implementation upon admission (or even upstream for certain patients) [25]—could be a response to Professor Hamburger.

Primarily, this proactive strategy serves to limit the risk of “medical excess” that may exist in the critical care environment [26]. It helps avoid futile interventions while reducing morbidity—for example, by preventing the onset of post-intensive care syndrome (PICS) [27] in patients whose severe comorbidities are known to dictate a poor long-term prognosis [27,28]. Parallel to this clinical benefit, such an approach also offers the advantages of safeguarding the future of critical care by improving the attractiveness of healthcare professions, reducing financial costs, and limiting environmental impacts, as suggested by our findings on GHG emissions.

More fundamentally, by aligning the principles of clinical relevance, economic sustainability, and ecological footprint, critical care medicine shifts, in actual practice, from a traditional obligation of means to an obligation of appropriate healthcare outcomes. Our findings on GHG emissions support this view and are underscored by the recent National [French] Academy of Medicine [5] report on environmental restraint in healthcare as a principle of civic solidarity. We hold that early ethical determination in the ICU represents an operational translation of this principle in practice.

## Conclusion

In critical care, a proactive ethical approach consisting of the early determination of the appropriate level of care for each patient—with the goal of avoiding non-beneficial treatment—appears to lead to a significant reduction in the GHG emissions of hospital stays. More broadly, such a course of action could constitute an essential element for the sustainability of critical care services.

## Author contributions

Conceptualization: PSP; Data curation: LM, RTS, DS, CAM, PSP, SR; Formal analysis: ML; Investigation: PSP, LM, SR; Methodology: CB, CL, JPQ, TM, ML, PSP; Project administration: PSP; Supervision: JPQ; Writing—original draft: PSP; Writing—review and editing: JPR, TM, ET, CB, JPQ.

## Consent for publication

Not applicable.

## Ethics approval and informed consent

Health data were collected post-hoc and de-identified, in accordance with French and European General Data Protection Regulations (GDPR). The study protocol received favorable institutional review board (IRB) approval from the French Intensive Care Society (SRLF) (IRB 00014135). Each patient (or their next of kin) was informed of the data collection for scientific and epidemiological purposes. Patients who expressed their refusal to participate (either directly or through their surrogates) to the use of their data were excluded from the study.

## Funding

This study received no external funding.

## Data availability

The data of the included patients are stored on a secure online server designed for observational studies (https://www.doqboard.com/). The data used to calculate the GHG emissions inventory are not confidential health data and are available in tabular format from the corresponding author.

## Declaration of competing interest

All authors have no competing interests to declare in any way related to this research.
