# Supplementary material for: The role of timely assessment of appropriate care in reducing greenhouse gas emissions in intensive care medicine
Source: Ann Intensive Care. 2026 Sep 17;16:100162. doi: 10.1016/j.aicoj.2026.100162 (PMC13634266; doi:10.1016/j.aicoj.2026.100162)
Supplement: Supplementary file 1 [file mmc1.docx]

**The Role of Timely Assessment of Appropriate Care in Reducing Greenhouse Gas Emissions in Intensive Care Medicine**

| **Category/Item** | **Emission Factor Unit** | **Value** | **SOURCE** |
| --- | --- | --- | --- |
|  |  |  |  |
| **ENERGY** | | | |
| Grid electricity | kgCO_2_eq / kWh | 0.032 | ADEME Carbon Database |
| Solar panel electricity | kgCO_2_eq / kWh | 0.055 | ADEME Carbon Database |
| Back-up generators | kgCO_2_eq / L of fuel oil | 3.25 | ADEME Carbon Database |
| Local heating plant (Chalon Énergie) | kgCO_2_eq / kWh | 0.122 | Local source^1^ |
| **TRANSPORTATION** | | | |
| Internal combustion engine (ICE) vehicle | kgCO_2_eq / km | 0.183 | ADEME Carbon Database |
| Electric vehicle | kgCO_2_eq / km | 0.11 | ADEME Carbon Database |
| Train | kgCO_2_eq / person.km | 0.026 | ADEME Carbon Database |
| Diesel bus | kgCO_2_eq / person.km | 0.04 | ADEME Carbon Database |
| Helicopter | kgCO_2_eq / L kerosene | 3.322 | ADEME Carbon Database |
| **PROCUREMENT OF GOODS AND SERVICES** | | | |
| Pharmaceuticals | kgCO_2_eq / € | 0.5 | ADEME Carbon Database |
| Medical devices | kgCO_2_eq / € | 0.315 | ADEME Carbon Database |
| Biology and imaging | kgCO_2_eq / exam | Per exam | Scientific literature^2, 3^ |
| Laundry services | kgCO_2_eq / € | 0.11 | ADEME Carbon Database |
| Meals | kgCO_2_eq / meal | 2.04 | ADEME Carbon Database |
| Water production | kgCO_2_eq / m^3^ | 6.03 | ADEME Carbon Database |
| Water treatment | kgCO_2_eq / m^3^ | 3.827 | ADEME Carbon Database |
| **CAPTIAL GOODS** | | | |
| Buildings | kgCO_2_eq / m^2^ | 440 | ADEME Carbon Database |
| Parking structures | kgCO_2_eq / m^2^ | 73 | ADEME Carbon Database |
| Biomedical equipment | kgCO_2_eq / € | 0.7 | ADEME Carbon Database |
| **WASTE TREATMENT** | | | |
| Non-hazardous waste | kgCO_2_eq / kg | 0.441 | ADEME Carbon Database |
| Clinical waste | kgCO_2_eq / kg | 0.934 | ADEME Carbon Database |
| Cardboard | kgCO_2_eq / kg | 0.047 | ADEME Carbon Database |

**SUPPLEMENTARY MATERIAL**

**Methodology Note and Reading Guide**

An emission factor (EF) represents an estimate (previously calculated through specific studies) of the quantity of GHG embedded in one unit of physical flow. These factors serve as the key to converting activity data measured during healthcare delivery into GHG quantities. The ADEME Carbon Database is a database hosted by ADEME (the French Agency for Ecological Transition) containing several thousand emission factors (<https://base-empreinte.ademe.fr/>).

Reading Guide: The consumption of one kWh of electricity from the French national grid results in the emission of 32g CO_2eq. The emission factor is therefore 0.032 kg CO_2eq/kWh.

EF = Emission Factor

^1^ These data were provided directly by the operator of the local district heating plant (City of Chalon-sur-Saône). The heat is generated from a mix of biomass and fossil gas. The value used represents the average value for the year 2023.

^2^ McGain F, Muret J, Lawson C, Sherman JD. Environmental sustainability in anaesthesia and critical care. Med J Aust. 2020;213(2):81-7. <https://doi.org/10.5694/mja2.50583>

^3^ McGain F, Sheridan N, Skillen K, et al. The carbon footprint of pathology testing. Lancet Reg Health West Pac. 2022;24:100459. <https://doi.org/10.1016/j.lanwpc.2022.100459>
